# Expression and Localization of CaBP Ca^2+^ Binding Proteins in the Mouse Cochlea

**DOI:** 10.1371/journal.pone.0147495

**Published:** 2016-01-25

**Authors:** Tian Yang, Elizabeth S. Scholl, Ning Pan, Bernd Fritzsch, Françoise Haeseleer, Amy Lee

**Affiliations:** 1 Department of Molecular Physiology and Biophysics, University of Iowa, Iowa City, Iowa, United States of America; 2 Department of Otolaryngology Head-Neck Surgery, University of Iowa, Iowa City, Iowa, United States of America; 3 Department of Neurology, University of Iowa, Iowa City, Iowa, United States of America; 4 Department of Biology, University of Iowa, Iowa City, Iowa, United States of America; 5 Department of Physiology and Biophysics, University of Washington, Seattle, Washington, United States of America; University of Washington, Institute for Stem Cells and Regenerative Medicine, UNITED STATES

## Abstract

CaBPs are a family of EF-hand Ca^2+^ binding proteins that are structurally similar to calmodulin. CaBPs can interact with, and yet differentially modulate, effectors that are regulated by calmodulin, such as Ca_v_1 voltage-gated Ca^2+^ channels. Immunolabeling studies suggest that multiple CaBP family members (CaBP1, 2, 4, and 5) are expressed in the cochlea. To gain insights into the respective auditory functions of these CaBPs, we characterized the expression and cellular localization of CaBPs in the mouse cochlea. By quantitative reverse transcription PCR, we show that CaBP1 and CaBP2 are the major CaBPs expressed in mouse cochlea both before and after hearing onset. Of the three alternatively spliced variants of CaBP1 (caldendrin, CaBP1-L, and CaBP1-S) and CaBP2 (CaBP2-alt, CaBP2-L, CaBP2-S), caldendrin and CaBP2-alt are the most abundant. By *in situ* hybridization, probes recognizing caldendrin strongly label the spiral ganglion, while probes designed to recognize all three isoforms of CaBP1 weakly label both the inner and outer hair cells as well as the spiral ganglion. Within the spiral ganglion, caldendrin/CaBP1 labeling is associated with cells resembling satellite glial cells. CaBP2-alt is strongly expressed in inner hair cells both before and after hearing onset. Probes designed to recognize all three variants of CaBP2 strongly label inner hair cells before hearing onset and outer hair cells after the onset of hearing. Thus, CaBP1 and CaBP2 may have overlapping roles in regulating Ca^2+^ signaling in the hair cells, and CaBP1 may have an additional function in the spiral ganglion. Our findings provide a framework for understanding the role of CaBP family members in the auditory periphery.

## Introduction

Calmodulin (CaM) is a member of a superfamily of Ca^2+^ binding proteins that are important transducers of Ca^2+^ signals controlling cellular excitability, gene expression, and the release of neurotransmitters and hormones [1]. These proteins have EF-hand domains, which are basic helix-loop-helix domains that upon binding Ca^2+^ ions, initiate a conformational change that modifies interactions with a variety of effectors [2]. For example, CaM in its Ca^2+^-free (apo) form associates constitutively with voltage-gated (Ca_v_) Ca^2+^ channels. Permeating Ca^2+^ ions bind to CaM, which triggers a negative feedback regulation of further Ca^2+^ entry through Ca_v_1 and Ca_v_2 channels (Ca^2+^-dependent inactivation, CDI, reviewed in [3]).

While CaM is expressed in all eukaryotic cells, some EF-hand Ca^2+^ binding proteins are expressed primarily in neuronal cell-types [4]. These include CaBPs, a family of Ca^2+^ binding proteins expressed in the brain, retina, and inner ear [5–9]. Like CaM, CaBPs can interact with Ca_v_ channels, but with distinct consequences. CaBPs inhibit CDI of Ca_v_1.2 and Ca_v_1.3 L-type channels [7, 8, 10–12]. This regulation by CaBPs may be important for the proper function of Ca_v_1.3 channels in mediating sustained glutamate release from inner hair cells in the cochlea. One CaBP family member, *CABP2*, has been identified as the locus for DFNB93 autosomal recessive hearing loss [13]. Compared to the wild-type CaBP2, the mutant form of CaBP2 is less able to suppress CDI of Ca_v_1.3 channels in transfected HEK293T cells [13]. The resulting increase in CDI would lead to reduced Ca_v_1.3 Ca^2+^ influx, which could impair transmission of auditory stimuli at the inner hair cell synapse.

In addition to CaBP2, three other CaBP family members have been detected by immunohistochemistry in inner hair cells (CaBP1, CaBP4, and CaBP5) [7–9]. However, genetic inactivation of CaBP4 in mice has nominal effects on CDI of Ca_v_1 channels in mouse inner hair cells, and has no effect on hearing [7]. These results could be explained by compensatory modulation of Ca_v_1.3 channels by the other CaBP family members. Alternatively, CaBP4 may be expressed at significantly lower levels than other CaBPs, and so may not play a major role in inner hair cells. Also unknown is whether hearing loss in humans that carry the *CABP2* mutation [13] results from loss-of function of CaBP2 in inner hair cells or in other auditory cell-types. Finally, CaBP1 and CaBP2 are subject to alternative splicing [5, 14, 15], but little is known about which CaBP splice variants are physiologically significant for hearing.

To begin to address these ambiguities, we characterized the relative levels and cellular localization of CaBPs in the mouse cochlea using quantitative reverse transcription PCR (qRT-PCR), *in situ* hybridization, and immunohistochemistry. Based on the mRNA levels, CaBP1 and CaBP2 are the major CaBPs in the cochlea. mRNA for CaBP1, but not for CaBP2, is up-regulated during development. CaBP1 is localized in hair cells and in cells resembling satellite glial cells in the spiral ganglion. By contrast, CaBP2 is strongly expressed in inner hair cells, and shows stronger expression than CaBP1 in outer hair cells during development. Our findings suggest a unique role for CaBP1 in the spiral ganglion and potentially overlapping roles for CaBP1 and CaBP2 in hair cells.

## Materials and Methods

### Animals

All experiments were performed in accordance with guidelines set by the Office of the Institutional Animal Care and Use Committee at the University of Iowa, which approved the animal use procedures used in this study, none of which were expected to produce pain or suffering in the animals. Wild type C57Bl/6 mice (Harlan Laboratories) were used for all experiments. Mice were housed in groups and maintained on a standard 12:12 hour light: dark cycle, with food and water provided *ad libitum*. Animals at postnatal day 21 (P21) were deeply anesthetized with isoflurane prior to sacrifice by decapitation. Cochleae were removed from the temporal bones within approximately 1–2 minutes and were flash-frozen in liquid nitrogen for total RNA extraction or were fixed by immersion in 4% paraformaldehyde (PFA) for *in situ* hybridization or immunofluorescent labeling.

### Quantitative RT-PCR

Total RNA was isolated from pairs of cochleae dissected from P7 or P21 C57Bl/6 mice (8 mice for each age) using TRIzol reagent (Life Technologies) combined with RNeasy RNA purification columns (Qiagen). Reverse transcription was performed using the SuperScript III First Strand Synthesis system (Life Technologies). Quantitative RT-PCR was performed using TaqMan gene expression assays and the StepOne Plus Real-Time PCR system. Catalog numbers for assays used were: Mm00600216_m1 (Cabp1, recognizes all splice variants); Mm01203518_m1 (Cabp1, recognizes CaBP1-long (CaBP1-L)); Mm01215744_m1 (Cabp2, recognizes all splice variants); Mm00489173_m1 (Cabp2, recognizes CaBP2-L); Mm01215748_m1 (Cabp2, recognizes CaBP2-S); Mm01215743_m1 (CaBP2, recognizes CaBP2-alt). In all experiments, assay Mm99999915_g1 for GAPDH was included as an endogenous control. The Life Technologies custom assay design tool was used to design qPCR assays specific for caldendrin and CaBP1-short (CaBP1-S). Specificity of assays for the intended targets was verified using plasmid DNAs. All assays were tested on serial dilutions of cochlear RNA and cDNA to verify that amplification efficiencies were similar. ΔC_T_ values were obtained from three technical replicates per cDNA sample. ΔC_T_ and RQ (relative quantity) values were calculated using the StepOne data analysis software. Data were averaged for 8 animals per age, with 2–3 qRT-PCR experiments (technical replicates) per animal. Error bars represent RQ values calculated from a 95% confidence interval of ΔC_T_ values. Statistical significance was determined using GraphPad Prism6 software.

### *In situ* hybridization

RNA probes for caldendrin were transcribed from PCR products from caldendrin plasmid DNA; probes that recognize all three isoforms of CaBP1 or CaBP2, or the CaBP2-alt isoform were transcribed from PCR products from mRNA extracted from the cochlea. The primers were: caldendrin F 5’- ATG AGC TCG CAC ATT GCC AAG AG-3’, R 5’- TAA CGA TCT GTC CTG GCC GAA G-3’; CaBP1 F 5;- GAG AGG CCA TGA GGA AGC TC-3’, R 5’- TGA GAT CAA AGG GCT AGG CG-3’; CaBP2-alt F 5’- GTG GTC CTG TGA AGG TTG GGA AGC-3’, R 5’- CTT CGA TCT CTT CTG GTC TCA GCT C-3’; CaBP2 F 5’- CTA CAT GCC CAC GGA GAT GG-3’, R 5’- GGA CGA GGT ATG GGG TGA TG-3’. Probes were labeled using a digoxigenin (DIG) RNA labeling kit (Roche). Whole mount *in situ* hybridization was performed according to established methods [16]. Cochleae from P6-P7 or P21 C57Bl/6 mice were fixed in 4% PFA and decalcified overnight in 4% PFA with 10% ethylenediaminetetraacetic acid (EDTA) in Phosphate Buffered Saline (PBS). Cochleae were digested with proteinase K (20 μg/ml, Ambion) for 30 minutes and incubated overnight at 60°C with sense or antisense RNA probes diluted in hybridization buffer (50% formamide and 50% 2x saline sodium citrate with 6% w/v dextran sulfate) with 10% denatured salmon sperm DNA (Life Technologies). Bound probes were detected using alkaline phosphatase-conjugated anti-digoxigenin antibody followed by detection with BM Purple substrate (Roche). Cochleae were dissected into apical, middle, and basal turns and were mounted in glycerol for imaging. As negative controls, the absence of labeling was verified in cochleae labeled with sense probes corresponding to each antisense probe (S1 Fig).

In some experiments, cochleae were processed for *in situ* hybridization and subsequently labeled with monoclonal myosin VIIa (Proteus Biosciences) as described previously [17]. DIG-labeled samples processed with BM Purple were re-fixed in 4% PFA in PBS, blocked in 2.5% normal goat serum with 0.5% Triton X-100, and incubated for 48 hours with antibody diluted in block solution. After washing 3 times in PBS (10 minutes each) at room temperature with agitation, samples were blocked again and incubated for 24 hours with Alexa 594- or 488-conjugated secondary antibody (Life Technologies) diluted in block solution. Cochleae were then dehydrated in ethanol and embedded in Poly/Bed 812 (Polysciences). Sections (10 μm) were cut using a Leica RM2265 microtome with glass knives and mounted on SuperFrost Plus slides (Fisher). Brightfield and fluorescence images were taken using an Olympus BX53 microscope and Olympus DP72 camera with CellSens Standard imaging software. Images corresponding to myosin VIIa / Alexa 488 labeling were false-colored red using Adobe Photoshop.

### Immunofluorescence

For whole mount staining, cochleae were dissected from C57Bl/6 mice at P7 or P20-23 and fixed for 1 hour in 4% PFA in PBS. Samples were then decalcified overnight in 1% PFA with 10% EDTA in PBS. Dissected cochleae were incubated for 60 minutes in blocking solution (5% normal goat serum and 0.5% Triton X-100 in 1x PBS) and then incubated at 4°C overnight with rabbit polyclonal antibody recognizing CaBP2 [5] and mouse monoclonal anti-myosinVIIa (Developmental Studies Hybridoma Bank, University of Iowa) diluted in blocking solution. After washing in PBS, samples were incubated for 1 hour in Alexa 488-conjugated goat anti-mouse and Alexa 647-conjugated goat anti-rabbit secondary antibodies (Life Technologies) diluted in blocking solution. Samples were washed in PBS and mounted using glycerol. Images were taken using an Olympus FluoView confocal laser scanning microscope with a 60x lens and FluoView software. Images represent optical sections taken at 1 μm intervals and compiled using the Z-stack function.

## Results

### *CaBP1* and *CaBP2* are the primary *CaBPs* expressed in the mouse cochlea

**Fig 1:**
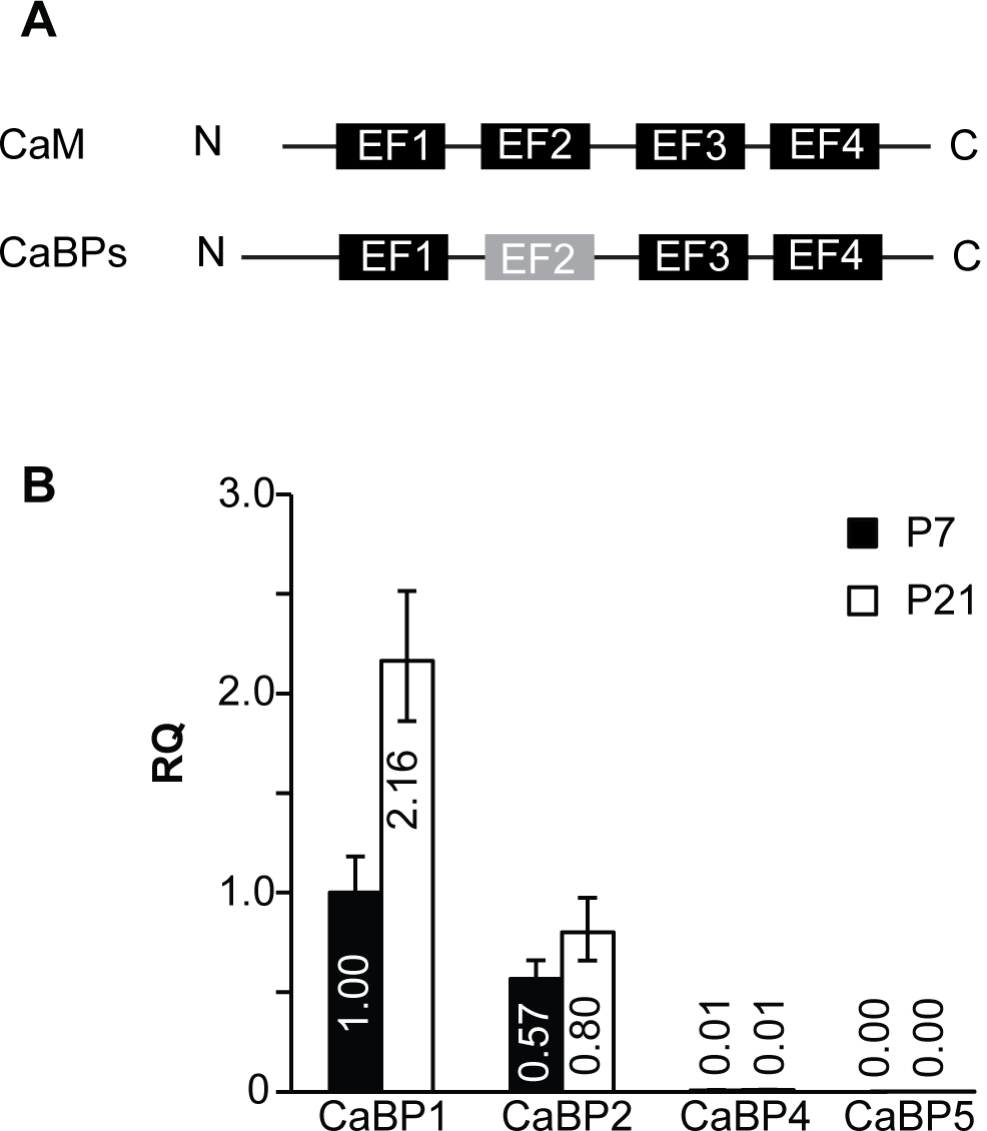
Analysis of CaBP1 and CaBP2 expression in mouse cochlea. qRT-PCR was performed using RNA isolated from mouse cochleae at P7 and P21. A) Schematic showing EF-hand domain structure of CaM and CaBPs with non-functional EF-2 indicated. B) Levels of total CaBP1 transcripts (CaBP1-L, CaBP1-S and caldendrin) and total CaBP2 transcripts (CaBP2-L, CaBP2-S, and CaBP2-alt). Data are expressed as relative quantities (RQ) compared to total CaBP1 transcripts at P7. Error bars indicate maximum and minimum RQ values calculated from a 95% confidence interval of ΔC_T_ values. n = 8 animals (16 cochleae) per age, 2–3 technical replicates per animal for each PCR assay.

Like CaM, CaBP1, 2, 4, and 5 have 4 EF-hand domains, the second of which is unlikely to bind Ca^2+^ due to key amino acid substitutions (Fig 1A; [4]). qRT- PCR was used to quantify the relative expression of CaBP mRNA transcripts in the mouse cochlea at developmental stages before (P7) and after (P21) the onset of hearing. Using TaqMan qPCR assays that recognize CaBP1, CaBP2, CaBP4 or CaBP5, we found that CaBP1 and CaBP2 are expressed at high levels, while CaBP4 and CaBP5 were barely detected (Fig 1B, Table 1). Between P7 and P21, CaBP2 levels did not change significantly, whereas CaBP1 levels nearly doubled (Fig 1B, Table 1).

**Table 1:** Comparison of CaBP expression levels in mouse cochlea at P7 and P21 by qRT-PCR.

	ΔCt values ± standard deviation		
	P7	P21	*p—value* (by t-test)
CaBP1	5.94±0.32	4.83±0.29	< 0.0001
CaBP2	6.76±0.28	6.26±0.37*	0.29
CaBP4	13.02±0.42^#^	12.82±0.38^#^	0.88
CaBP5	19.72±1.51^#^	17.84±0.75^#^	< 0.05

ΔCt values were determined as described in Materials and Methods.

*, p <0.01

^#^, p <0.0001 compared to CaBP1 at same age (by one-way ANOVA and post-hoc Bonferroni’s multiple comparisons test). For each assay, n = 8 biological replicates.

**Fig 2:**
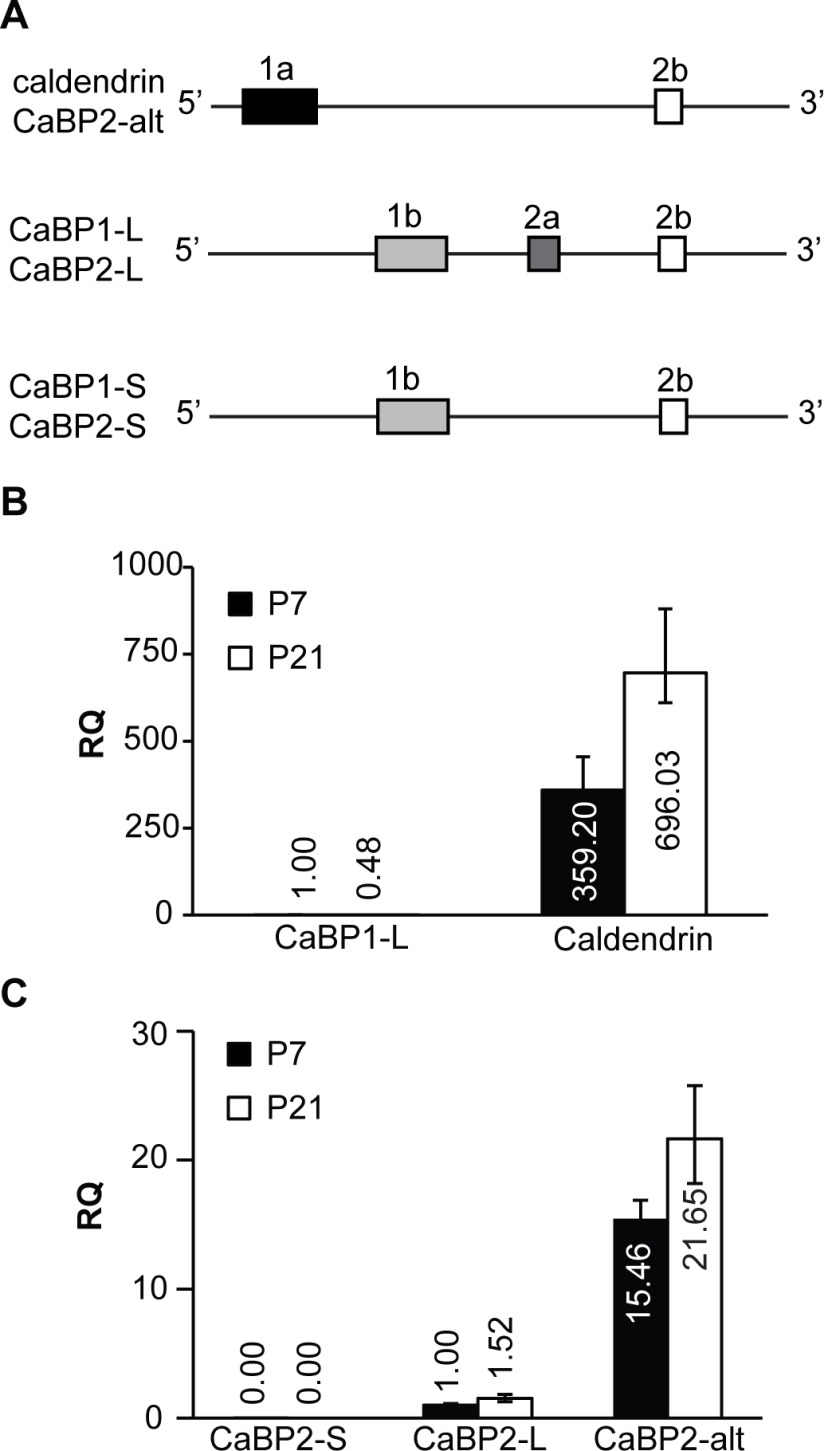
Analysis of CaBP1/2 splice variant expression in mouse cochlea. A) Schematic showing splice variants of CaBP1 and CaBP2 with differences in the N-terminal region. Although encoded by separate genes, CaBP1 and CaBP2 undergo similar patterns of alternative splicing of exons 1a, 1b, 2a, and 2b. B) Levels of CaBP1-L and caldendrin variants, expressed relative to levels of CaBP1-L at P7. C) Levels of CaBP2-S, CaBP2-L, and CaBP2-alt variants, expressed relative to CaBP2-L at P7. (B-C) Numbers indicate relative quantities. Error bars indicate maximum and minimum RQ values calculated from a 95% confidence interval of ΔC_T_ values. n = 7 animals (14 cochleae) for caldendrin and CaBP1 at P7; n = 8 animals (16 cochleae) per age for the rest of the assays; 2–3 technical replicates per animal for each PCR assay.

Alternative splicing gives rise to three variants of CaBP1 (CaBP1-S, CaBP1-L, and caldendrin) and CaBP2 (CaBP2-alt, CaBP2-S, and CaBP2-L), which differ in their N-terminal sequences [5, 14, 15]. Caldendrin utilizes a exon 1a, while CaBP1-S and CaBP1-L have exon 1b. CaBP1-L differs from caldendrin and CaBP1-S in containing exon 2a (Fig 2A). In the brain, caldendrin is expressed at higher levels than CaBP1-S/L [18]. Although encoded by a different gene, CaBP2 undergoes a similar pattern of alternative splicing as CaBP1. Like caldendrin, CaBP2-alt contains a distinct N-terminal domain due to inclusion of exon 1a, while exon usage of CaBP2-S and CaBP2-L is similar to that of CaBP1-S and CaBP1-L, respectively (Fig 2A). To determine whether CaBP1 and CaBP2 splice variants are differentially expressed in the cochlea, we performed qRT-PCR experiments using TaqMan assays specific to single variants. TaqMan assays specific to CaBP1-S were not available. Therefore, we focused on caldendrin and CaBP1-L. These experiments revealed that caldendrin is the predominant CaBP1 variant expressed in the cochlea, with levels ~300–700 times greater than those of CaBP1-L at P7 and P21 (Fig 2B, Table 2). Similar to total levels of all CaBP1 variants (Fig 1B), caldendrin levels approximately double between P7 and P21 (Fig 2B, Table 2). Quantitation of CaBP2 transcripts revealed that CaBP2-alt expression is ~15–22 times higher than that of CaBP2-L; CaBP2-S was barely detected (Fig 2C, Table 2). Like caldendrin (Fig 2B), CaBP2-alt underwent a significant although more modest developmental increase in expression (Fig 2C, Table 2). These results indicate that caldendrin and CaBP2-alt are the dominant CaBP variants expressed in the mouse cochlea and are developmentally regulated.

**Table 2:** Comparison of CaBP splice variant expression levels in mouse cochlea at P7 and P21 by qRT-PCR.

	ΔCt values ± standard deviation		
	P7	P21	*p—value* (by t-test)
Caldendrin	6.78±0.24 (n = 7)	5.83±0.25	<0.001
CaBP1-L	15.27±0.26* (n = 7)	16.32±0.58*	<0.05
CaBP2-alt	3.79±0.17	3.31±0.34	<0.05
CaBP2-L	7.74±0.23^#^	7.14±0.36^#^	<0.05
CaBP2-S	23.03±1.15^#^	Not detected	--

ΔCt values were determined as described in Materials and Methods.

*, p <0.0001 compared to caldendrin at same age

^#^, p <0.0001 compared to CaBP2-alt at same age by one-way ANOVA and post-hoc Bonferroni’s multiple comparisons test. N = 7 biological replicates for Caldendrin and CaBP1-L at P7, and n = 8 biological replicates for the rest of the tests.

### *CaBP1/Caldendrin* is expressed in the spiral ganglion and the organ of Corti

**Fig 3:**
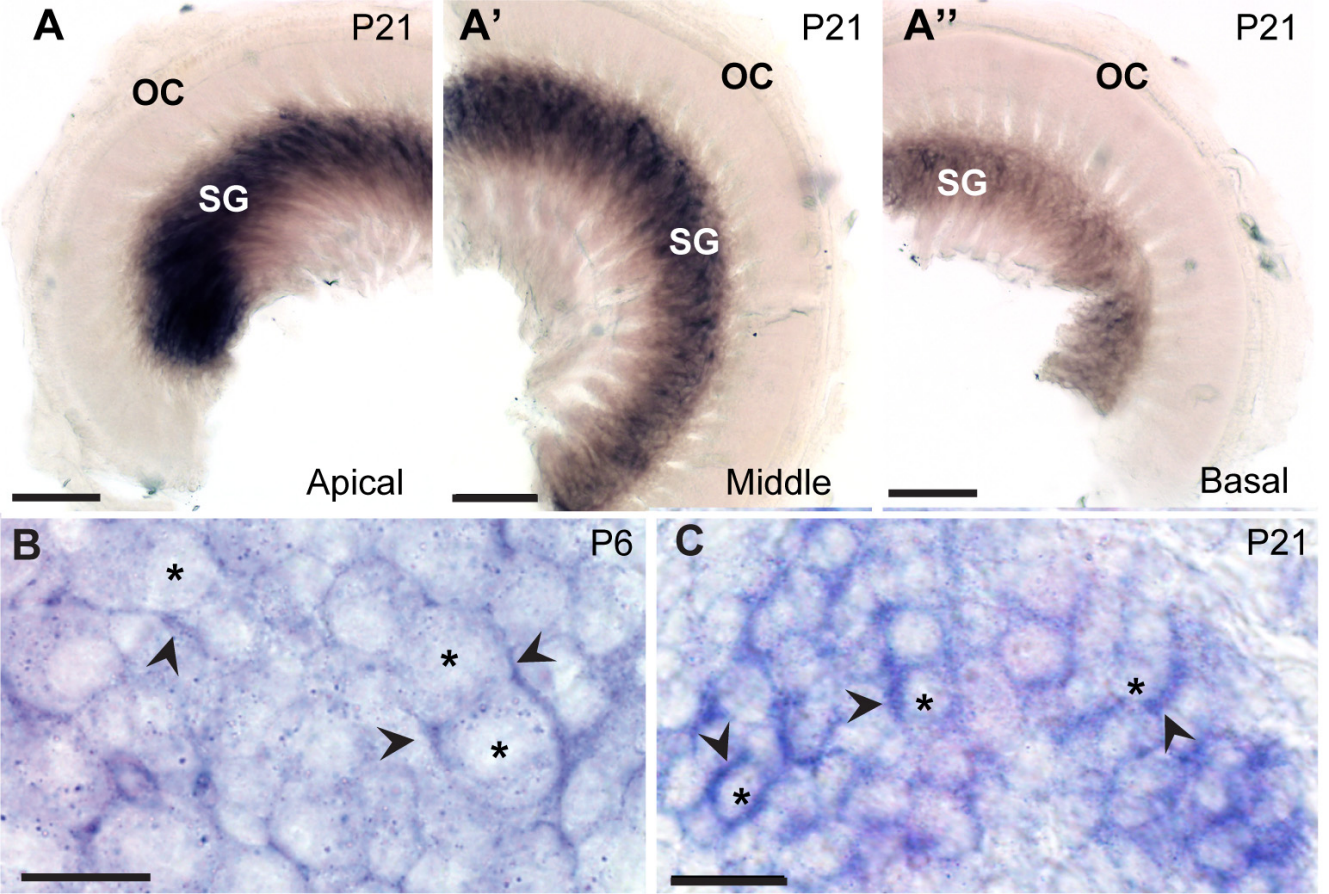
*In situ* hybridization probes against caldendrin label the spiral ganglion of the mouse cochlea. The RNA probe was designed to recognize exon 1a of the *Cabp1* gene that is unique to caldendrin. (A-A”) Caldendrin *in situ* hybridization in mouse cochlea at P21in the apical (A), middle (A’) and basal (A”) turns of P21 mouse cochlea. OC, organ of Corti; SG, spiral ganglion. (B,C) 10 μm-thick plastic sections showing caldendrin *in situ* hybridization in mouse cochlea at P6 (B) and P21 (C). Asterisks indicate spiral ganglion neuron cell bodies; arrowheads indicate strong caldendrin signal in cells around neuron cell bodies. Scale bars: 100 μm (A-A”); 10 μm (B); 20 μm (C).

Given that caldendrin was the most abundant CaBP in the mouse cochlea according to our qRT-PCR analyses, we performed *in situ* hybridization experiments to determine its cellular localization. An RNA probe was designed against *Cabp1* exon 1a, which encodes the long N-terminal sequence specific to caldendrin. At P21, caldendrin mRNA was detected in the spiral ganglion, with little to no signal in the organ of Corti (Fig 3A–3A”). Caldendrin labeling was the strongest in the apical turn (Fig 3A) and gradually lessened in the middle (Fig 3A’) and basal turns (Fig 3A”). To analyze the subcellular localization of caldendrin, labeled cochleae were cut into 10 μm-thick plastic sections, which yielded greater cellular detail compared to whole-mount sections. Unexpectedly, strong hybridization signal was seen surrounding, but not within, neuronal cell bodies (Fig 3B and 3C).

**Fig 4:**
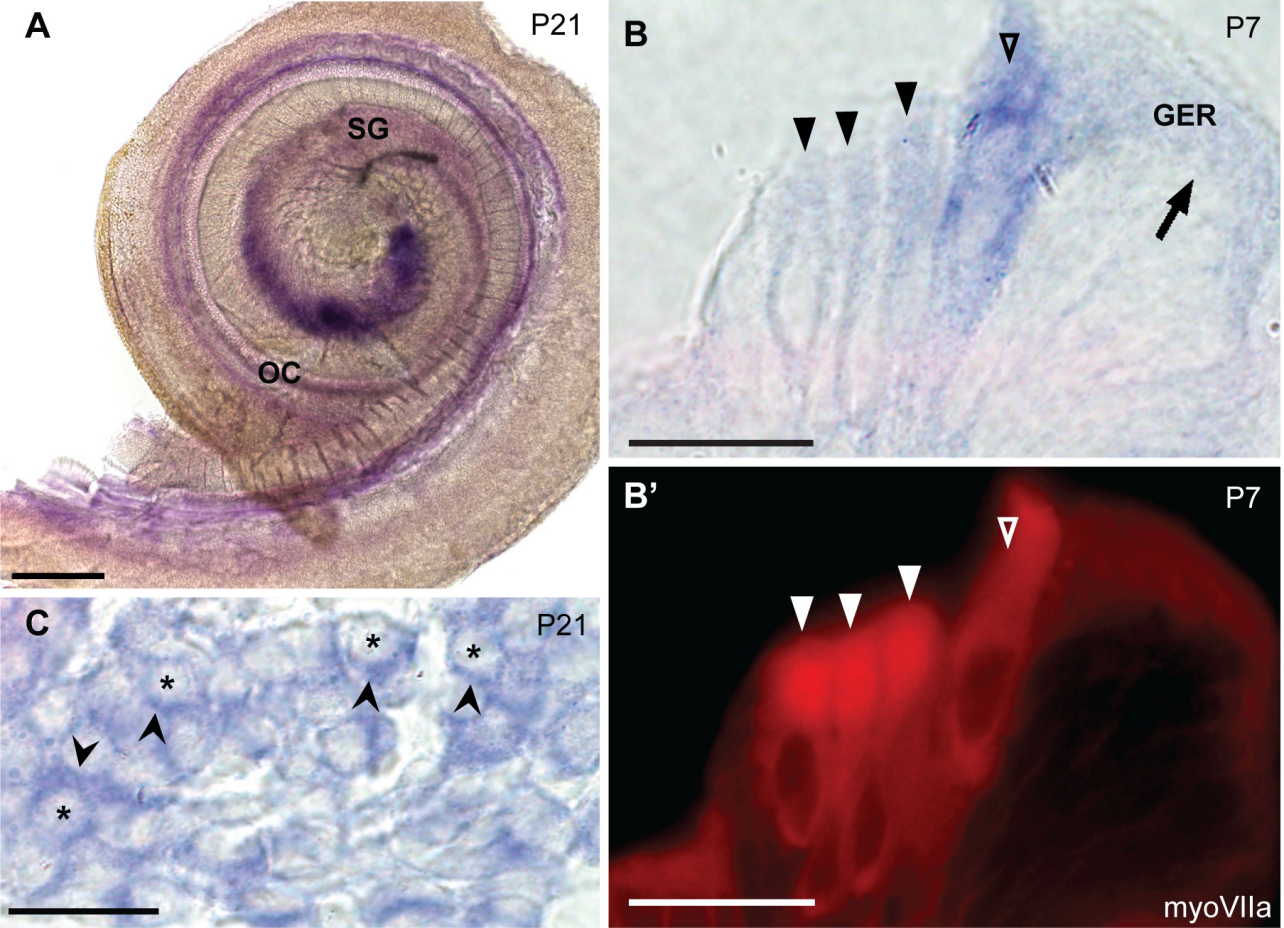
*In situ* hybridization probes against caldendrin, CaBP1-L, and CaBP1-S label hair cells and the spiral ganglion. The pan-CaBP1 probe was designed to recognize the 3’ end of the *Cabp1* gene such that it should recognize all three variants of CaBP1. (A) *In situ* hybridization of a P21 mouse cochlea, showing pan-CaBP1 labeling in the spiral ganglion (SG) and the organ of Corti (OC). (B-B’) 10 μm-thick plastic section showing *in situ* hybridization signal in the organ of Corti (P7) double labeled with antibodies against myosin VIIa. Open triangles indicate inner hair cells; filled triangles indicate outer hair cells; arrow indicates the inner supporting cells in greater epithelial ridge (GER). (C) 10 μm-thick plastic section showing expression of CaBP1/caldendrin *in situ* hybridization in the spiral ganglion. Asterisks indicate spiral ganglion neuron cell bodies; arrowheads indicate pan-CaBP1 signal in cells surrounding neuron cell bodies. Scale bars: 200 μm (A); 20 μm (B, C).

We next asked whether the other two CaBP1 variants, CaBP1-L and CaBP1-S, show a similar localization to that of caldendrin. Due to the limited sequence differences between the 3 variants, it was not possible to generate probes that would recognize CaBP1-S and CaBP1-L but not caldendrin. Therefore, we designed a probe targeting the 3’ end of the CaBP1 mRNA that was designed to recognize all three isoforms (pan-CaBP1). With this probe, we reasoned that any signal outside of the spiral ganglion would represent CaBP1-S/L. At P21 (Fig 4A), pan-CaBP1 labeling was detected in the spiral ganglion, similar to that for caldendrin (Fig 3A–3A”). However, weak pan-CaBP1 labeling was also present in the organ of Corti (Fig 4A, 4B and 4B’). The localization of the pan-CaBP1 labeling in hair cells was confirmed by immunofluorescent labeling with antibodies against myosin VIIa (Fig 4B–4B’). In 10 μm-thick plastic sections, pan-CaBP1 labeling appeared to be associated primarily with inner hair cells although weaker labeling was detectable in outer hair cells (Fig 4B and 4B”), and the apical surfaces of the inner supporting cells in the greater epithelial ridge (Fig 4B). As with the caldendrin-specific probe (Fig 3B and 3C), the strong pan-CaBP1 labeling in the spiral ganglion was associated with cells surrounding the neurons, which resembled satellite glial cells (Fig 4C). These results support the cell-type specific expression of CaBP1 variants, with caldendrin primarily expressed in the spiral ganglion and CaBP1-L and/or CaBP1-S expressed in the hair cells in the Organ of Corti.

### *CaBP2* is highly expressed in hair cells

**Fig 5:**
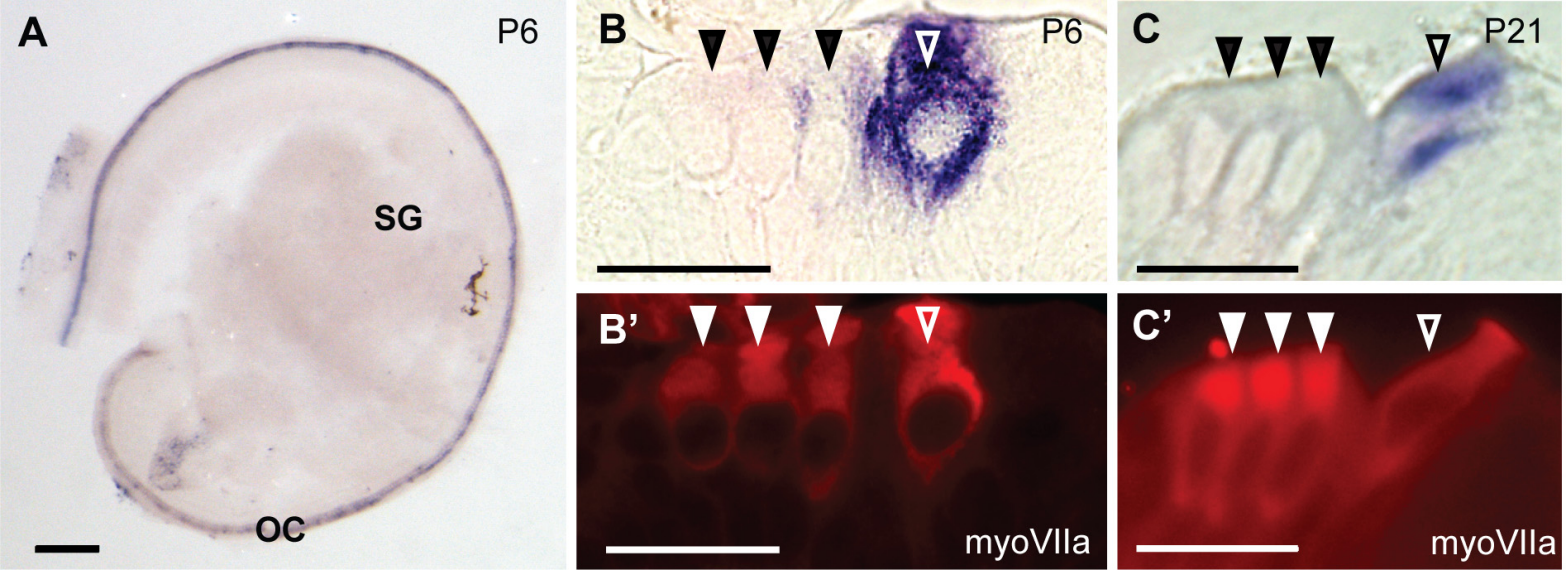
*In situ* hybridization probes against CaBP2-alt label cochlear hair cells. The RNA probe was designed to recognize exon 1a unique to the CaBP2-alt splice variant. (A) CaBP2-alt *in situ* hybridization signal in a mouse cochlea at P6. (B-C’) 10 μm-thick plastic sections of mouse cochlea at P6 (B) or P21 (C) were hybridized with CaBP2-alt *in situ* probe and labeled with antibodies against myosin VIIa (myoVIIa) for immunofluorescence. CaBP2-alt *in situ* hybridization signal (B,C) is localized to myoVIIa-labeled inner hair cells (B’, C’). Filled triangles indicate outer hair cells; open triangles indicate inner hair cells. Scale bars: 200 μm (A); 20 μm (B-C’). OC, organ of Corti; SG, spiral ganglion.

Because CaBP2-alt was the primary CaBP2 splice variant expressed in the mouse cochlea, we performed *in situ* hybridization using an RNA probe targeted against exon 1a encoding the unique sequence in the CaBP2-alt N-terminus. In contrast to caldendrin, CaBP2-alt labeling was specifically localized within the organ of Corti at P6 (Fig 5A) and P21 (data not shown). To confirm that CaBP2-alt is expressed in hair cells, the CaBP2-alt labeled cochleae were immunofluorescently labeled with myosin VIIa antibodies and then cut into 10 μm-thick plastic sections. At both P7 and P21, CaBP2-alt labeling was intense in inner hair cells (Fig 5B–5C’). CaBP2-alt labeling between hair cells likely emanates from inner hair cells out of the main focal plane, although we cannot discount that it represents expression in supporting cells.

**Fig 6:**
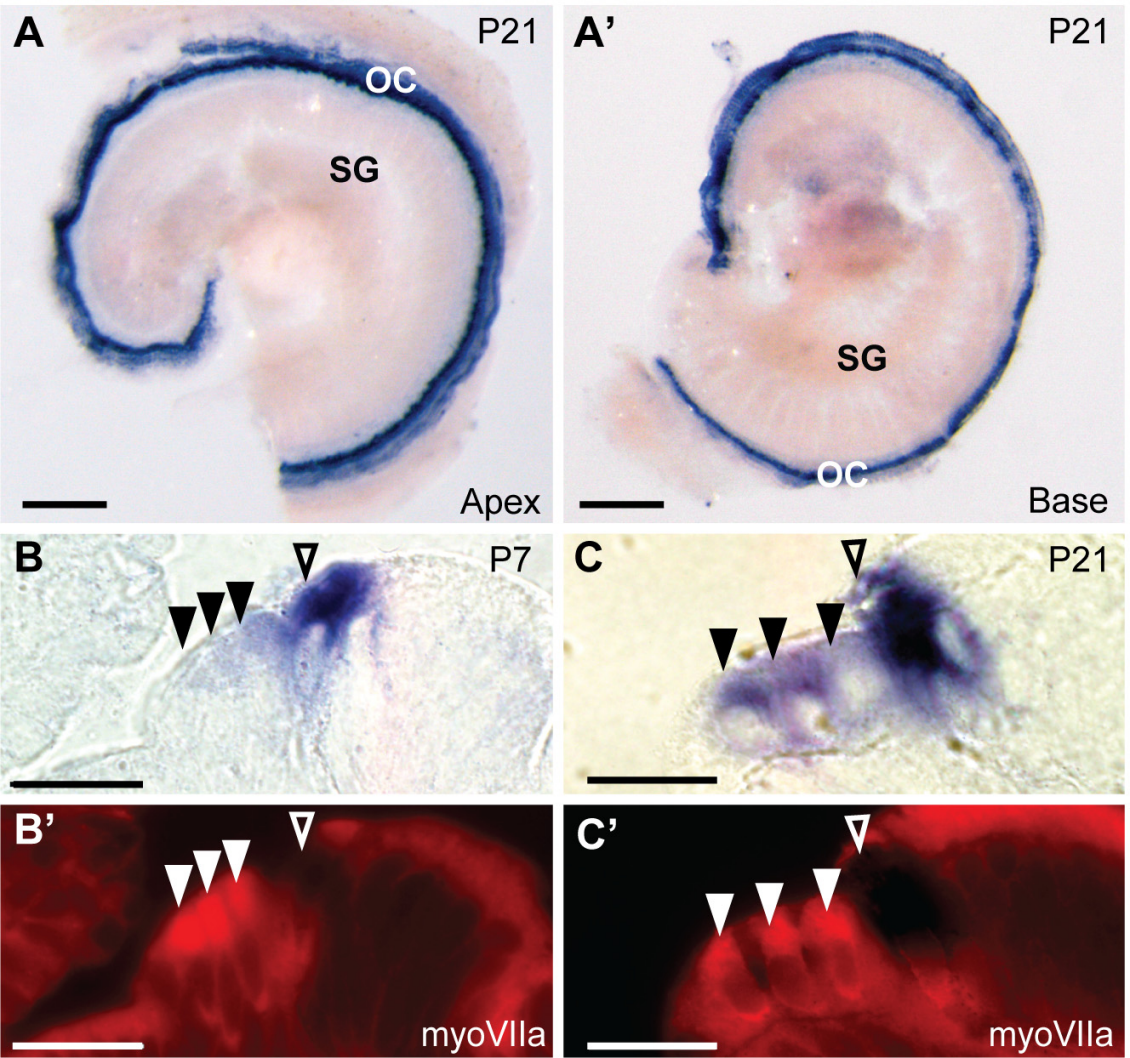
*In situ* hybridization probes against CaBP1-alt, CaBP2-L and CaBP2-S label inner and outer hair cells. The RNA probe was designed to recognize all splice variants of CaBP2. (A-A’) *In situ* hybridization signal in the apical (A) and basal (A’) turn of a mouse cochlea at P21. (B-C’) 10 μm-thick plastic sections of cochlea hybridized with pan-CaBP2 *in situ* probe and immunofluorescently labeled with antibodies against myosin VIIa (myoVIIa) immunofluorescence at P7 (B) or P21 (C). Images represent in situ hybridization signal (A,A’, B,B’) or myoVIIa immunofluorescence (B’,C’). Filled triangles indicate outer hair cells; open triangles indicate inner hair cells. Scale bars: 200 μm (A-A’), 20 μm (B-C’). OC, organ of Corti; SG, spiral ganglion.

We also designed a pan-CaBP2 probe that targets the 3’ end of CaBP2 mRNA and would recognize all three CaBP2 variants. In contrast to distinct labeling patterns produced by the caldendrin and pan-CaBP1 probes (Figs 2 and 3), labeling with the pan-CaBP2 probe was similar to that of CaBP2-alt in that it was abundant in the organ of Corti at P7 (data not shown) and P21 at both the apex and base of the cochlea (Fig 6A and 6A’). We examined pan-CaBP2 labeling in 10 μm-thick plastic sections that were double-labeled with antibodies against myosin VIIa. At P7, similar to results obtained with the CaBP2-alt probe (Fig 5B and 5C), pan-CaBP2 labeling was strong in inner hair cells, and weak in the first row of outer hair cells (Fig 6B). Unlike labeling for CaBP2-alt, which was not seen in outer hair cells (Fig 5), pan-CaBP2 labeling was detected in all three rows of outer hair cells at P21 (Fig 6B and 6C). These results suggest that CaBP2-S and/or CaBP2-L, but not CaBP2-alt, is weakly expressed in outer hair cells prior to hearing onset and upregulated after hearing onset. Since CaBP2-S was barely detected by qRT-PCR (Fig 2C, Table 2), the pan-CaBP2 labeling of outer hair cells likely represents CaBP2-L expression.

**Fig 7:**
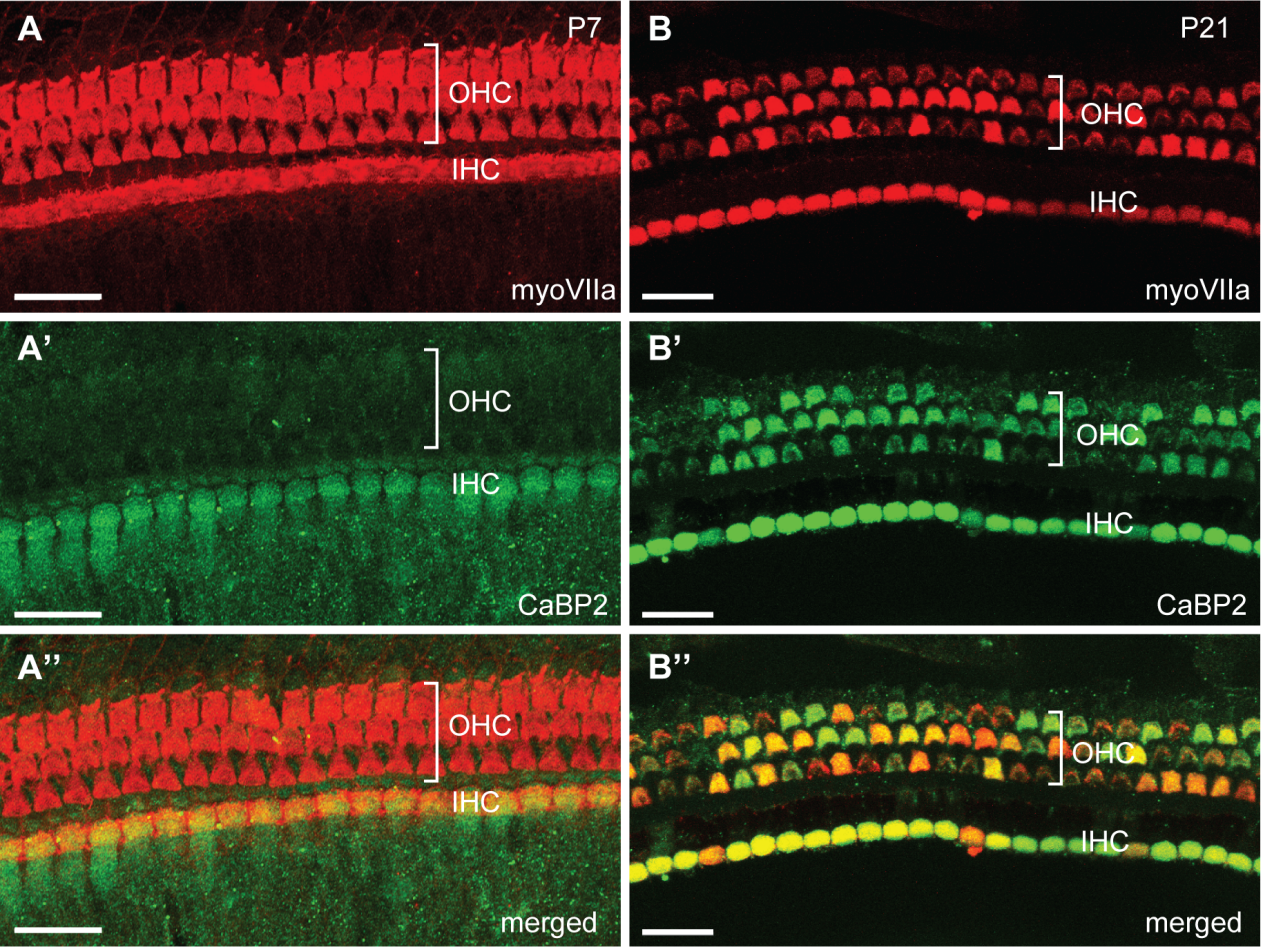
Stronger immunolabeling for CaBP2 in outer hair cells after hearing onset. Immunocytochemistry was performed using antibodies against CaBP2 and MyoVIIa on P7 and P21 mouse cochlea. Images show labeling for MyoVIIA (A,B), CaBP2 (A’,B’), or both in the merged images (A”,B”). At P7 (A-A”), CaBP2 is detected in the inner hair cells (IHCs). At P21 (B-B”), CaBP2 is detected in inner hair cells and outer hair cells (OHCs). For both P7 and P21, results are shown for the apical turn of the cochlea. Patchy labeling is likely an artifact of the light fixation conditions required for CaBP2 antibody labeling, since it was reproduced in 3 independent experiments. Scale bars: 20 μm.

Compared to labeling at P7, labeling of outer hair cells with the pan-CaBP2 probe was stronger at P21 (Fig 6B and 6C). In agreement with this result, immunolabeling with CaBP2 antibodies recognizing all three CaBP2 variants was strong in outer hair cells at P21 compared to P7 (Fig 7). Apparently, the CaBP2 antibodies are not sensitive enough to detect protein expression corresponding to the weak CaBP2-L mRNA levels in outer hair cells at P7 (Fig 5B). Consistent with our *in situ* hybridization results (Fig 6A–6D), CaBP2 antibodies strongly labeled both inner and outer hair cells at P21 (Fig 7). Thus, at both the mRNA and protein levels, the expression of CaBP2 in outer hair cells may be subject to developmental regulation and is greater after hearing onset.

## Discussion

In this study, we characterized the expression levels and localization of CaBPs in the cochlea. We show that CaBP1 and CaBP2 are the major CaBPs expressed in the cochlea and that they have different expression patterns. Moreover, the splice variants of both CaBP1 and CaBP2 exhibit differences in cellular localization. Our results provide a foundation for future studies of the molecular targets and physiological roles of CaBP family members in auditory function.

### CaBP1 and CaBP2 as modulators of Ca_v_1.3 channels in hair cells

A proposed role for CaBPs in the auditory system was based initially on findings that Ca_v_1.3 channels, which are crucial for inner hair cell function [19], are directly modulated by CaBPs in transfected HEK293T cells [7, 8]. In contrast to the properties of Ca_v_1.3 channels expressed in other cell-types, Ca_v_1.3 channels in inner hair cells exhibit little CDI [8, 20–22]. Hence the identification of CaBPs as major suppressors of CDI [10] led to further analyses of whether CaBPs contributed to the native properties of Ca_v_1.3 in inner hair cells [7, 8]. While previous immunohistochemistry studies have shown CaBP4 and CaBP5 immunolabeling of inner and outer hair cells [7, 8], our results argue against a major role for these CaBPs in regulating Ca_v_1.3 in inner hair cells. By qRT-PCR, CaBP4 and CaBP5 mRNA were barely above the level of detection (Fig 1B; Table 1). The previous detection of CaBP4 and CaBP5 by immunohistochemistry may be due to the signal amplification afforded by this technique and variable specificities of the different CaBP antibodies [7, 8]. It is also possible that the turnover rates of CaBP4 and CaBP5 protein are very slow such that their levels may be greater than would be expected based on their levels of mRNA.

Our results also indicate that caldendrin is unlikely to modulate Ca_v_1.3 channels in inner hair cells, since it is not expressed in the organ of Corti (Fig 3). While the pan-CaBP1 probe labeled hair cells at P7 and P21 (Fig 4), the signal was weak compared to that for CaBP2 (Figs 5 and 6). Since labeling with the caldendrin-specific probe was not seen in hair cells (Fig 2), pan-CaBP1 labeling in hair cells likely represents CaBP1-S and/or CaBP1-L, which could therefore contribute to the regulation of Ca_v_1.3 in inner hair cells. Our results are consistent with a previous transcriptomic analysis of mouse outer and inner hair cells, which showed that while mRNA for CaBP4 and CaBP5 was barely detectable, mRNA levels for CaBP1 and CaBP2 were moderate and strong, respectively [23].

Our initial study did not indicate a significant effect of CaBP2 on CDI of Ca_v_1.3 channels in a heterologous expression system [7]. However, we subsequently showed that at higher expression levels, CaBP2 showed robust suppression of CDI of Ca_v_1.3 channels [13]. The specific and intense expression of CaBP2 in inner hair cells (Figs 5,6 and 7) is highly consistent with a role as Ca_v_1.3 regulator. The mutation associated with DFNB93 causes truncation of the C-terminal EF-hands of CaBP2, which inhibits its ability to modulate Ca_v_1.3 channels. This effect would limit synaptic Ca^2+^ influx, which would presumably impair inner hair cell transmission to spiral ganglion afferents. The mutation causes moderate-to-severe hearing loss [13]. The lack of a more severe hearing phenotype in these patients may be due to partial compensation by CaBP1. Hearing loss associated with DFNB93 is most severe in the mid-frequency range [13]; although we did not observe tonotopic differences in CaBP2 expression (Figs 5 and 6), such gradients could exist in humans. Ca_v_1.3 Ca^2+^ channels are also expressed in outer hair cells [24, 25], where they regulate exocytosis that can activate type II afferent neurons [26]. In the immature mouse (<P10), CDI of Ca_v_1.3 channels is stronger in outer hair cells compared to inner hair cells [19, 24], which could be explained by the weak expression of CaBP2 in outer hair cells at this age (Figs 6 and 7). Taken together, our results support a role for CaBP2 as a likely modulator of Ca_v_1.3 channels in inner and outer hair cells, which may account for hearing impairment associated with DFNB93.

### A possible role for caldendrin in satellite glial cells

In the brain, caldendrin is associated with the postsynaptic density of excitatory synapses [18]. Thus, we had anticipated that caldendrin expression in the spiral ganglion corresponded to a neuronal localization. Surprisingly, caldendrin probes labeled the cells surrounding the spiral ganglion neurons, possibly satellite glial cells based on their morphology (Fig 3B and 3C, Fig 4C). However, we cannot rule out a low level of expression of caldendrin in the spiral ganglion neurons that is beyond the detection limit of *in situ* hybridization. CaBPs have been considered to be largely neuron-specific [5], although caldendrin has been detected in the acrosome of sperm cells [27, 28]. The function of the satellite glial cells in the spiral ganglion is largely unknown, but based on their roles in other sensory ganglia [29], these cells may regulate the microenvironment of spiral ganglion neurons. Like CaBP1, caldendrin enhances the opening of Ca_v_1.2 Ca^2+^ channels by suppressing CDI [30]. Immunofluorescence studies show that multiple Ca_v_ Ca^2+^ channels, including Ca_v_1.2, are present in glial cells within the spiral ganglion [31] and in other parts of the peripheral nervous system [32]. The role of Ca_v_1.2 channels in these glial cells remains unclear. However, Ca_v_1 channel-mediated Ca^2+^ entry promotes myelination by oligodendrocytes [33]. The possibility that caldendrin participates in processes regulating myelination of spiral ganglion cells by satellite glial cells is intriguing. Considering that it has similar effects on Ca_v_1.2 channels to those of CaBP1 [30], caldendrin may regulate targets known to be modulated by CaBP1. For example, CaBP1 regulates inositol 1,4,5-trisphosphate receptors [34–36], which promote the release of neurotrophins from satellite glial cells [37]. Thus, caldendrin may have diverse roles in regulating Ca^2+^ signaling in satellite glial cells in the spiral ganglion through interactions with plasma membrane and intracellular Ca^2+^ channels.

### Significance of *CaBP* splice variants

A question that emerges from our study is regarding the importance of multiple CaBP splice variants. Currently, there is limited evidence that alternative splicing of CaBPs significantly affects their functional interactions with targets. The alternatively spliced N-terminal regions of CaBP1 and CaBP2 are upstream of the first EF-hand domain and thus far have not been shown to differentially impact function. For example, CaBP1-S/L and caldendrin can both inhibit CDI of Ca_v_1.2 channels, although to varying extents [30]. However, CaBP1-S and CaBP1-L have been found to exhibit differences in their subcellular localization in transfected cells [5]. Moreover, the distinct localization of CaBP1-L/S and caldendrin in the organ of Corti and the spiral ganglion supports different roles of these variants in auditory function. An understanding of how CaBP splice variation influences Ca^2+^ signaling and the physiological functions of CaBPs remains an important challenge for future studies.

## Supporting Information

S1 Fig Lack of *in situ* hybridization signal for CaBPs in mouse cochlea using sense probes. *In situ* hybridization was performed in whole mounts of mouse cochlea (P21) using sense mRNA probes corresponding to caldendrin (A), all three variants of CaBP1(B), CaBP2-alt (C), and all three variants of CaBP2 (D) on P21 cochleae. OC, organ of Corti; SG, spiral ganglion.
(DOCX)
