# Supplementary material for: Expression and Localization of CaBP Ca2+ Binding Proteins in the Mouse Cochlea
Source: PLoS One. 2016 Jan 25;11(1):e0147495. doi: 10.1371/journal.pone.0147495 (PMC4725724; doi:10.1371/journal.pone.0147495)
Supplement: S1 Fig — In situ hybridization was performed in whole mounts of mouse cochlea (P21) using sense mRNA probes corresponding to caldendrin (A), all three variants of CaBP1(B), CaBP2-alt (C), and all three variants of CaBP2 (D) on P21 cochleae. OC, organ of Corti; SG, spiral ganglion. (DOCX) [file pone.0147495.s001.docx]

**Supporting information**


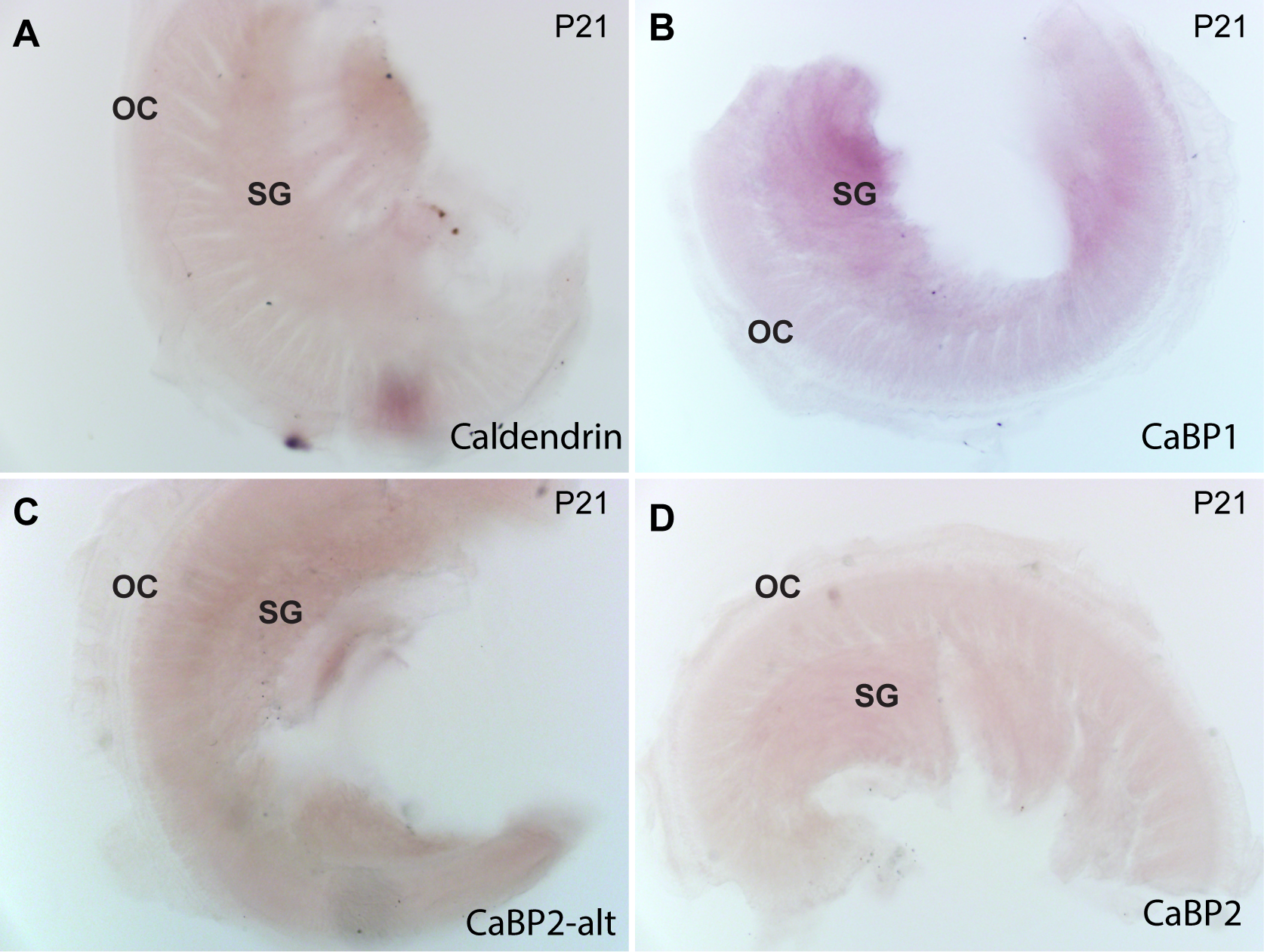


**Figure S1: Lack of *in situ* hybridization signal for CaBPs in mouse cochlea using sense probes.**

*In situ* hybridization was performed in whole mounts of mouse cochlea (P21) using sense mRNA probes corresponding to caldendrin (A), all three variants of CaBP1(B), CaBP2-alt (C), and all three variants of CaBP2 (D) on P21 cochleae. OC, organ of Corti; SG, spiral ganglion.
